# A Longitudinal Study: Changes in Cortical Thickness and Surface Area during Pubertal Maturation

**DOI:** 10.1371/journal.pone.0119774

**Published:** 2015-03-20

**Authors:** Megan M. Herting, Prapti Gautam, Jeffrey M. Spielberg, Ronald E. Dahl, Elizabeth R. Sowell

**Affiliations:** 1 Department of Pediatrics, Keck School of Medicine at USC/Children’s Hospital of Los Angeles, Los Angeles, California, United States of America; 2 Neuroimaging Research for Veterans Center, VA Boston Health Care System, Boston, Massachusetts, United States of America; 3 Institute of Human Development, University of California, Berkeley, California, United States of America; Hôpital Robert Debré, FRANCE

## Abstract

Sex hormones have been shown to contribute to the organization and function of the brain during puberty and adolescence. Moreover, it has been suggested that distinct hormone changes in girls versus boys may contribute to the emergence of sex differences in internalizing and externalizing behavior during adolescence. In the current longitudinal study, the influence of *within-subject* changes in puberty (physical and hormonal) on cortical thickness and surface area was examined across a 2-year span, while controlling for age. Greater increases in Tanner Stage predicted less superior frontal thinning and decreases in precuneus surface area in both sexes. Significant Tanner Stage and sex interactions were also seen, with less right superior temporal thinning in girls but not boys, as well as greater decreases in the right bank of the superior temporal sulcus surface area in boys compared to girls. In addition, within-subject changes in testosterone over the 2-year follow-up period were found to relate to decreases in middle superior frontal surface area in boys, but increases in surface area in girls. Lastly, larger increases in estradiol in girls predicted greater middle temporal lobe thinning. These results show that *within-subject* physical and hormonal markers of puberty relate to region and sex-specific changes in cortical development across adolescence.

## Introduction

Puberty marks the onset of adolescence, with dramatic increases in the sex hormones: estradiol (E_2_) in girls and testosterone (T) in boys [1,2]. Hormonal changes in puberty lead to advancements of primary and secondary sexual characteristics, which typically begin to develop earlier in girls (~age 10) compared to boys (~age 11.5) [3,4,5,6]. Recent research suggests that pubertal development not only affects physical sexual maturity, but may also influence cortical maturation and may do so in a sex-specific fashion [7,8]. However, studies in this area are typically cross-sectional and/or dichotomize individuals based on physical markers of puberty, which limits examination of how *individual differences* in pubertal maturation influence brain development during adolescence. The goal of the present longitudinal study was to characterize how pubertal changes relate to cortical maturation in girls and boys during this key developmental window.

Dividing gray matter volume (obtained via magnetic resonance imaging (MRI)) into two morphometric components (cortical thickness, surface area) allows for better quantification of cortical maturation [9]. Using these metrics, total cortical thickness and surface area show distinct, sex-specific trajectories across childhood and adolescence [10,11]. Given the differences in trajectories, it is feasible that distinct neurobiological factors contribute to each of these morphometric components. Interestingly, animal studies suggest that sex and puberty have wide effects on cellular composition that may contribute differentially to cortical thickness and surface area. Specifically, sex differences have been reported in apoptosis in the visual cortices [12] as well as in maturation of dendritic branching and spines [13,14,15]. During puberty, T contributes to new cell growth patterns within medial temporal lobe structures [16], whereas E_2_ has been shown to inhibit cortical myelination [17]. Together, these findings suggest that pubertal maturation and its associated sex-steroids may influence cortical structure differentially in girls and boys.

To our knowledge, only three studies have examined longitudinally how puberty or hormones impact cortical thickness [18,19,20], and no study in this area has examined surface area. Specifically, two studies examined how androgens (T and didehydroepiandrosterone (DHEA)) relate to cortical thickness using a longitudinal sample between the ages of 4 to 22 years [19,20]. In girls, an inverted-U relationship was seen with T-related thickening of somatosensory cortices during childhood, whereas T predicted thinning in early adulthood. In contrast, in post-pubertal boys, higher levels of T predicted less cortical thickness in the posterior cingulate and the dorsal lateral prefrontal cortex [19]. For DHEA, higher levels predicted increases in cortical thickness at younger, pre-pubertal ages in both sexes [20]. However, since both studies examined a wide age-range and split individuals into pre- and post-puberty groups, sex differences in pubertal development in cortical thickness remains unclear.

In the third longitudinal study, cortical thickness was linked to androgen receptor signaling efficiency [18]. Higher efficiency was related to region-specific increases or decreases in cortical thinning rates in boys, whereas it predicted only thinning in girls. Notably, these longitudinal studies focused only on androgens. Only three studies have been published on E_2_ and brain structure in adolescent girls, all of which were cross-sectional [21,22,23]. Therefore, it is unclear how *within-subject* change in E_2_ influences cortical trajectories during puberty, or how physical maturation or sex hormones relate to longitudinal changes in surface area.

The current study utilizes a longitudinal design and multiple measures of pubertal maturation in order to characterize in more detail how physical and hormonal (E_2,_ T) changes during puberty relate to cortical thickness and surface area development. A number of additional analyses utilizing the longitudinal sample utilized in this report have previously been published using functional MRI [24,25] as well as different morphologic MRI measures [26] obtained from this dataset (also see below). The longitudinal design for the sample included collecting neuroimaging and pubertal maturation indices within the same girls and boys at two time points, ~2 years apart. Because girls begin to display physical markers of puberty 1–2 years earlier than boys [3,4], the initial ages of the sample recruited was 10 to 12 years in girls (mean = 11.84±.74) and 12 to 14 years in boys (mean = 12.93±.68). This longitudinal study design with a relatively restricted age range allowed us to capture pre-pubertal stages in both sexes (10–14 years in girls and 12–16 years in boys), which would not be feasible if the groups were originally recruited matched by age. Furthermore, by examining within-subject data, we have the unique ability to determine how changes in pubertal maturation over the two-year interval affect cortical development between the sexes.

Previous research utilizing different imaging modalities in this same longitudinal sample have focused on structural MRI development in subcortical structures, based on a body of research suggesting that adolescence is a particularly important period in subcortical developmental [27]. Both T and E_2_ evidenced complex relationships with subcortical structural volume in this same dataset [26]. Functional imaging studies in the current sample have also found that increases in T are associated with greater subcortical functional reactivity to threat in both amygdala and ventral striatum [24] as well as T-dependent changes in functional coupling between amygdala and orbitofrontal cortex [25]. In addition to subcortical structures, changes in total gray and white matter volume in the current longitudinal sample found that gray matter volume was related to E_2_ but not T [26]. Given that only total volume was examined, specific region(s) driving this finding is unknown. Furthermore, it is feasible that the previous examination of total gray matter volume might have obscured region-specific relationships with T. Thus, past research in this sample and others [18,19,20] hint at puberty-related changes in cortical thickness and surface area. Furthermore, we hypothesized that sex differences in cortical thickness and surface area would emerge as a function of pubertal maturation.

## Material and Methods

### Data collection, analysis, and data availability

R.E.D. and colleagues at the University of Pittsburgh collected all data, whereas M.M.H. and E.R.S. conducted structural MRI preprocessing and data analyses at Children’s Hospital of Los Angeles. Time 1 cross-sectional assessment of structural MRI data as well as longitudinal analysis of subcortical volumes in this sample has been previously reported [26,28,29]. Given that the data used for this manuscript involved human participants, data use (including de-identified basic demographic information and brain thickness and surface area values for the significant clusters) will be considered for qualified researchers upon request, and upon institutional review board approval from all proposed research sites. Requests should be submitted to the Developmental Cognitive Neuroscience Laboratory at brainstudy@chla.usc.edu.

### Ethics statement

All participants and their legal guardians provided informed written consent to participate in the current study as approved by the University of Pittsburgh Institutional Review Board. The current research was also conducted according to the principles expressed in the Declaration of Helsinki. Children’s Hospital Los Angeles (CHLA) Institutional Review Board approved the data analyses to be performed at CHLA (IRB# CCI-11-00191).

### Participants

126 (63 girls) typically developing adolescents, ages 10 to 14, were recruited through advertisements, flyers, and demographically targeted phone lists. Exclusionary criteria for all participants included a lifetime diagnosis of psychiatric disorders, irremovable metal, history of head injury, serious medical illness, or psychotropic medication. Due to attrition (n = 31; 52% girls; 42% due to braces) and poor MRI image acquisition due to motion (n = 8 at Time 1 and n = 6 at Time 2), longitudinal data was successfully obtained for 33 boys for cortical thickness and 32 boys for surface area, whereas 48 girls had usable cortical thickness and surface area estimates. A breakdown of the data for each time point by sex can be found in Table 1.

**Table 1:** Puberty measurements in boys and girls.

		Boys (n = 33*)			Girls (n = 48)	
		Time 1	Time 2		Time 1	Time 2
Tanner Stage	n = 28	2.70 ± .98	4.27 ± 1.00	n = 31	2.73 ± .88	4.56 ± .74
Testosterone (ng/mL)	n = 23	150.42 ± 118.48	363.48 ± 135.87	n = 33	44.92 ± 12.20	59.09 ± 14.64
Estradiol (pg/mL)		—	—	n = 36	35.71 ± 16.75	89.09 ± 58.88

Values represent mean and standard deviation

*Denotes n = 32 for surface area

### Pubertal maturation

Tanner Staging of physical maturation was determined by a trained research nurse practitioner using criterion based on Marshall and Tanner [30]. A finger-stick procedure was used to collect blood samples [31]. Given the circadian and sleep influences on hormone levels, all blood samples were obtained between 8:20 and 8:35 AM. It is important to acknowledge the challenges in capturing and controlling for the cyclical changes in hormone variations in girls during pubertal maturation given that variability in menstrual cycle length is greatest 1–2 years following menarche [32] and approximately 80% of girls are often anovulatory in the first year after menarche [33,34]. Efforts were made to reduce hormone variations in post-menarchal girls by collecting data during follicular phase. However, given that the majority of post-menarchal girls were studied approximately 6 months after the onset of menarche (range of menarche date at time 2 was 0 to 11 months prior to the study), their cycles were highly variable in length and irregular. Thus, although a number of efforts were made to control for cycle phase, irregular menses made it difficult to be certain with precision where individual girls were in their cycles.

Once collected, blood samples were used to assess free index values of sex-steroids (T in boys; T and E_2_ in girls) via modification of commercially available serum/plasma radioimmunoassay kits (T: DSL/Beckman Coulter; E_2_: Siemens, Los Angeles, CA). Sensitivity measured as the minimum detectable dose (MDD) and inter-assay coefficients of variation (CV) for low, medium, and high BioRad external controls for T were MDD = .04 ng/mL; CV = 7.2% (low), 11.4% (medium) and 4.3% (high). None of the subjects were below the minimum sensitivity thresholds, indicating the assays were sensitive to T in pre-pubertal females and males. E_2_ sensitivity and inter assay coefficients of variation were also acceptable, with the Siemens BS-serum regression curve of y = 2.7056 (pg/mL). Due to a manufacturing discontinuation of products, hormone data could not be established for all subjects. Of the 33 boys, 28 had Tanner Stage and 23 had T data for both time points. Of the 48 girls, 31 had Tanner Stage, 33 had T and 36 had valid E_2_ data for both visits. Means and standard deviations for each of the measures per time point and sex can be found in Table 1. For each index of pubertal maturation (Tanner Stage, E_2_, T), maturational change (pubertal Δ) was calculated as (Time 2—Time 1)/(scan interval), representing the amount of maturation that occurred over the follow-up interval.

### Structural imaging acquisition

Whole-brain T1-weighted MPRAGE images were acquired for each participant at Time 1 and Time 2 using a 3-Tesla Siemens Allegra scanner. Scan parameters for the MPRAGE were as follows: repetition time (TR) = 1540 ms; echo time (TE) = 3.04 ms; flip angle = 8°; field of view = 256x256; voxel = 1mm^3^.

### Image processing and statistical analyses

Statistical analyses were carried out in R and FreeSurfer. Thickness and surface area estimates were determined using FreeSurfer’s longitudinal stream, which allows for estimates that are unbiased with respect to any time point [35]. The longitudinal preprocessing stream includes: 1) processing of all time points separately using the cross-sectional pipeline (e.g. removal of non-brain tissue, image registration to Talairach space, segmentation of ICV into gray, white, and CSF tissue, subcortical parcellation, intensity normalization, spatially smoothed with a Gaussian kernel with a full width half maximum of 10 mm)[36,37], 2) the creation of a probabilistic template for each participant that is unbiased to either time point, 3) using the cross-sectional stream to process each participant’s unbiased template, and 4) then re-processing of each time point using the unbiased template. This last step allows for the unbiased template to be utilized in several processing steps including skull stripping, Talairach transformations, atlas registration, and ultimately significant increases in reliability and statistical power when estimating volume segmentations [35]. Each of these preprocessing steps were manually checked by MMH who was blind to participant demographics (sex, age, pubertal status) and scan session.

For the current study, whole brain vertex-based primary cortical thickness dependent variables of interest included: the average (in mm)[average = 0.5* (Time 1 thickness + Time 2 thickness)] and rate of change (in mm/year)[rate = (Time 2 thickness—Time 1 thickness)/(Time 2—Time 1)]. Therefore, positive rates of change in thickness are interpreted as growth, whereas negative rates of change are interpreted as thinning. Similarly, surface area dependent variables of interest included the average (in mm^2^)[average = 0.5* (Time 1 surface area + Time 2 surface area)] and rate of change (in mm^2^/year)[rate = (Time 2 surface area—Time 1 surface area)/(Time 2—Time 1)].

FreeSurfer’s Qdec program was used to perform whole brain vertex-based analyses for the dependent variables of interest, including average cortical thickness, cortical thickness change rate, surface area, and surface area change rate. For each measurement of puberty (Tanner Stage; T; E_2_), the influence of pubertal Δ (i.e. Tanner Stage Δ; T Δ; or E_2_ Δ), sex, and their interactions on average cortical thickness (or surface area) was examined, while controlling for age, puberty (Tanner Stage, T, or E_2_) at Time 1, and scan interval. Similarly, for each measurement of puberty (Tanner Stage; T; E_2_), the influence of pubertal Δ, sex, and their interactions on cortical thickness rate (or surface area rate) was examined, while controlling for age and puberty at Time 1. Scan interval was not included in the rate of change models as it was already accounted for in the calculation of the dependent variable (thickness or surface area rate of change). To correct for multiple comparisons, Monte Carlo corrections were applied, and significance was determined as those regions with *p-values <*.*01* after correction.

## Results

### Puberty measures

Raw values of within-subject changes in Tanner Stage, T, and E_2_ are presented in S1 Fig. At Time 1, a large portion of both girls and boys had pre-pubertal Tanner Stage values (Stages 1–2), whereas as at Time 2 the majority of each sex was at mid-to-late puberty (Stages 3–5) as by study design (S1 Fig.). Mixed model analyses showed that age significantly predicted Tanner Stage (Age: *β* = .72, *p*<.0001), but this did not vary as a function of sex (Age-by-Sex Interaction: *β* = .07, *SE* = .10, *p* = .52). Age was also found to significantly predict T in boys (Age: *β* = 99.32, *SE* = 9.4, *p*<.0001) as well as T (Age: *β* = 6.16, *SE* = .84, *p*<.0001) and E_2_ (Age: *β* = 21.33, *SE* = 3.53, *p*<.0001) in girls. As expected, T levels varied depending on sex, with boys having higher values and larger increases in levels with age (Sex: *β* = 107, *SE* = 7.8, *p*<.0001; Age-by-Sex Interaction: *β* = -93.28, *SE* = 7.8, *p*<.0001). Thus, in order to remove sex-related differences in the mean and variance of T scores, T values were converted to z-scores separately within each sex.

### Puberty and brain maturation

#### Tanner stage

**Fig 1:**
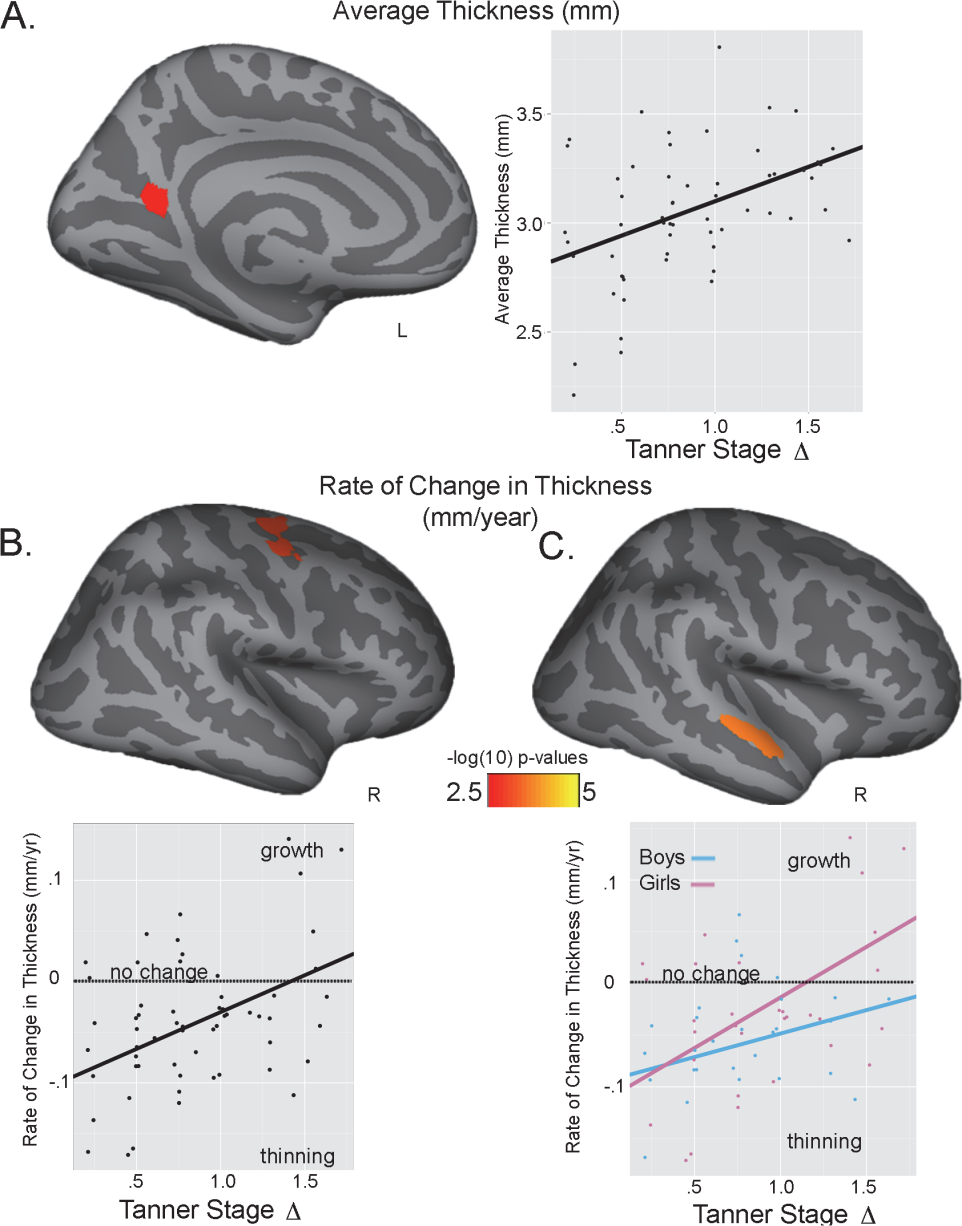
Tanner Stage and thickness. A.) Main effect of Tanner Stage Δ on average thickness (mm) in the left cuneus. Plot reflects the raw data with the fit line of the main effect. B) Main effect of Tanner Stage Δ on rate of change in thickness (mm/year) in the right superior frontal gyrus. Plot reflects the raw data with the fit line of the main effect. C.) Tanner Stage Δ-by-sex interactions seen for rate of change in thickness (mm/year) in the right superior temporal gyrus. Plot reflects raw data with the fit lines of the interaction effect; blue line = boys; pink line = girls. In B & C plots the dotted line reflects no change, with positive values (above line) reflecting growth and negative values (below line) reflecting thinning.

**Fig 2:**
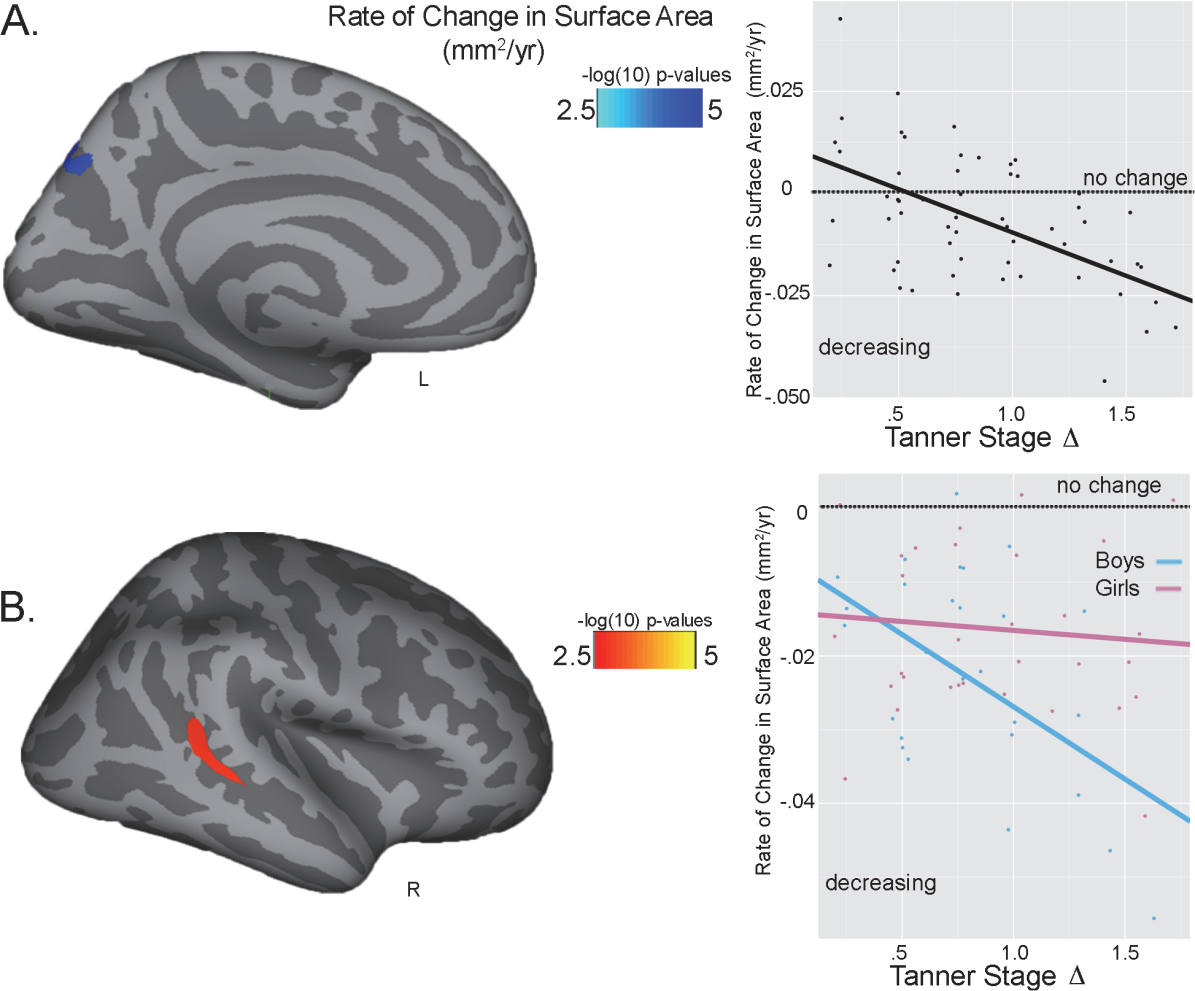
Tanner Stage and surface area. A.) Main effect of Tanner Stage Δ on rate of change in surface area (mm^2^/yr) in the left precuneus. Plot reflects the raw data with the fit line of the main effect; dotted line reflects no change, with negative values (below line) reflecting larger decreases in surface area. B.) Tanner Stage Δ-by-sex interaction seen for rate of change in surface area (mm^2^/yr) in the right banks of the superior temporal sulcus. Plot reflects raw data with the fit lines of the interaction effect; blue line = boys; pink line = girls; negative values reflecting larger decreases in surface area.

A significant main effect for Tanner Stage maturation was seen for average cortical thickness of the left cuneus, with greater Tanner Stage Δ predicting a thicker cortex similarly in both sexes at follow-up (Tanner Stage Δ: partial η ^2^ = .180) (Fig. 1). A significant effect of Tanner Stage maturation was also seen for rate of thickness change, with greater Tanner Stage Δ predicting less cortical thinning at follow-up in the right superior frontal cortex for both sexes (Tanner Stage Δ: partial η ^2^ = .041) (Fig. 1). A significant Tanner Stage Δ-by-sex interaction was also detected in the right superior temporal gyrus. Greater Tanner Stage Δ predicted less thinning, with a larger effect in girls than boys (Tanner Stage Δ-by-sex: partial η ^2^ = .034) (Fig. 1). For surface area, both a Tanner Stage Δ main effect and a significant Tanner Stage Δ-by-sex interaction were found for rate of change in surface area. In both sexes, greater Tanner Stage Δ was related to decreases in left precuneus surface area (Tanner Stage Δ: partial η ^2^ = .041) **(** Fig. 2). However, in the right banks of the superior temporal sulcus, greater Tanner Stage Δ related to significant decreases in surface area in boys, but not in girls (Tanner Stage Δ-by-sex: partial η ^2^ = .079) (Fig. 2). No relationship was seen between Tanner Stage and average surface area.

### Testosterone (T)

**Fig 3:**
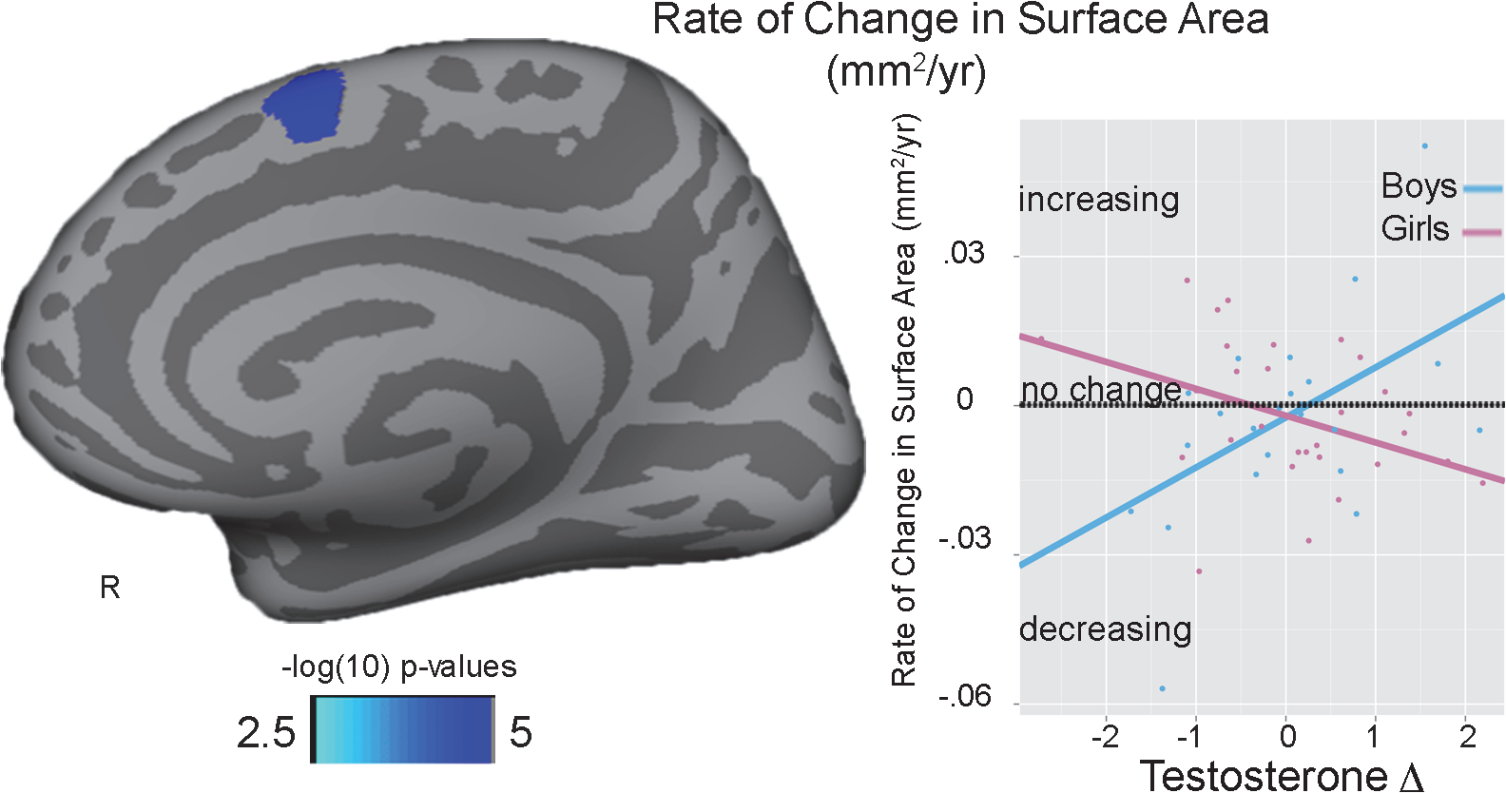
Testosterone and surface area. T Δ-by-sex interaction seen for rate of change in surface area (mm^2^/yr) in the right medial superior frontal cortex. Plot reflects raw data with the fit lines of the; blue line = boys; pink line = girls; dotted line reflects no change, with positive values (above line) reflecting larger increases and negative values (below line) reflecting larger decreases in surface area.

A significant T Δ-by-sex interaction was seen for the rate of change in right medial superior frontal surface area, with larger T Δ relating to increases in surface area in boys but decreases in surface areas in girls (T Δ-by-sex: partial η ^2^ = .223) (Fig. 3). Changes in T (T Δ) did not predict average cortical thickness, rate of thickness change, or average surface area in either sex.

### Estradiol (E_2_) in girls

**Fig 4:**
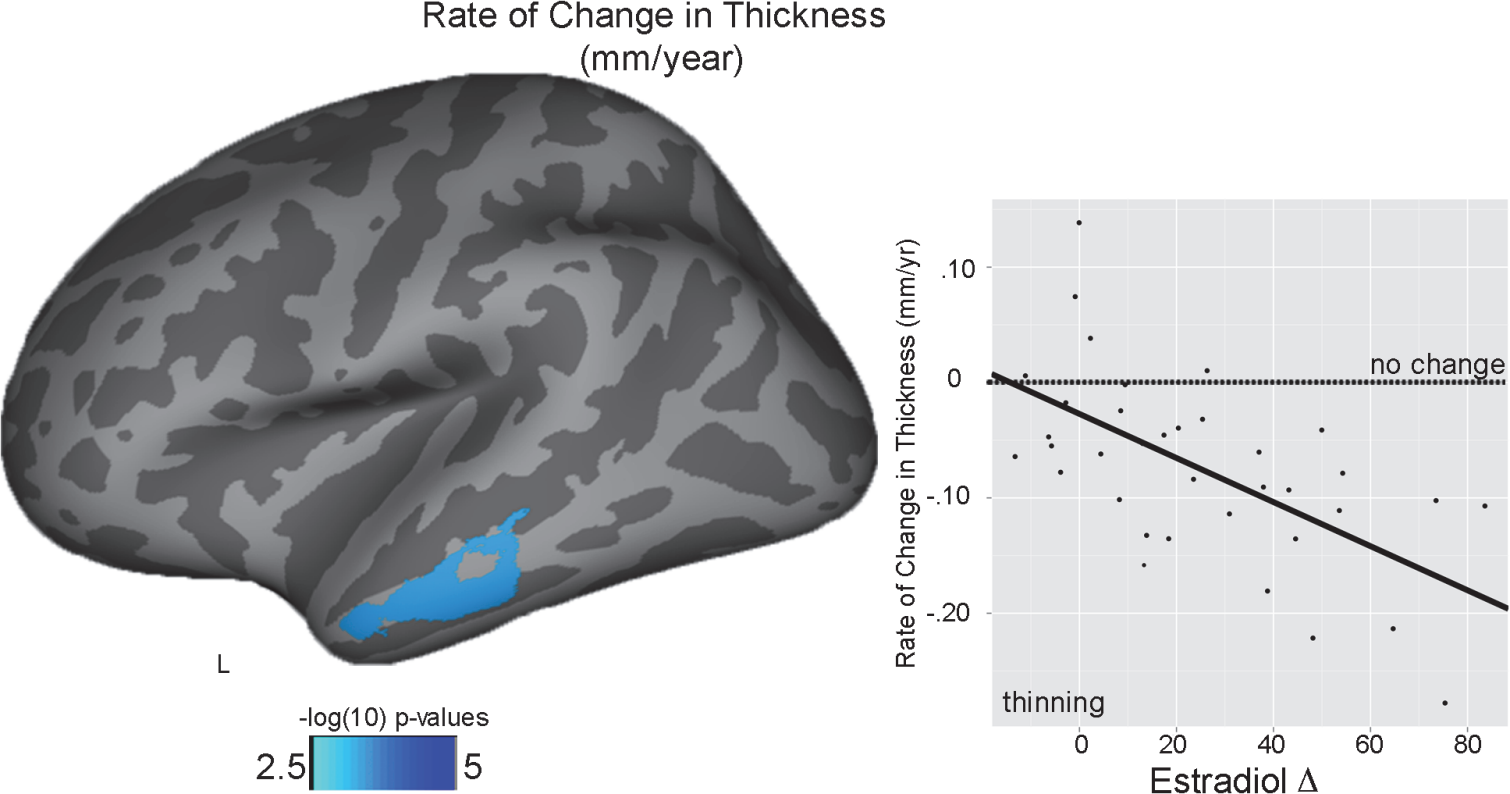
Estradiol and thickness in girls. Main effect of E_2_ Δ on cortical rate of change in thickness (mm/year) in the left superior temporal gyrus. Plot reflects the raw data with the fit line of the main effect; dotted line reflects no change, with negative values (below line) reflecting thinning.

For rate of change in cortical thickness, greater changes in E_2_ Δ at follow-up predicted greater thinning of the left middle temporal gyrus (E_2_ Δ: partial η ^2^ = .434) (Fig. 4). Changes in E_2_ did not significantly relate to average thickness, average surface area, or rate of change in surface area.

## Discussion

Sex differences in structural brain development emerge during adolescence and may be driven, in part, by puberty-specific processes [7,8,38]. By studying pre-pubertal girls and boys at Time 1 and then following the same individuals over time, in the current study we were able to capture a wide-range of individual differences in the increases in sex hormones during puberty over a ~2 year developmental ‘age’ period. These individual differences in hormone changes, in turn, were found to predict cortical thickness and surface area maturation. We show that, independent of age, greater physical maturation across a 2-year period predicted decreased superior frontal thinning for both sexes, but only girls showed this relationship in superior temporal cortex. We also describe, for the first time, how within-subject change in Tanner Stage and T relate to decreases in surface area across sex in the precuneus, as well as in a sex-specific fashion in portions of the frontal and temporal lobes. Furthermore, we show that larger increases in E_2_ across the 2-year period resulted in greater left middle temporal gyrus thinning in girls. These findings are important as they suggest that individual differences in the change of sex hormones during puberty predict cortical brain maturation.

Our findings for T and E_2_ and cortical thickness expand on previous cross-sectional findings [21,22,29]. T predicted sex-specific changes in medial superior frontal surface area, with greater individual increases in T relating to decreases in surface area in girls, but increases in boys. Individual differences in T-related changes in the maturation of the medial superior frontal cortex are in agreement with the findings of Nguyen and colleagues [19] who found a negative association between T and cortical thickness in post-pubertal girls in a similar region. In contrast to the longitudinal study by Nyguen and colleagues, we did not detect a significant relationship between T and cortical thickness. There are a number of reasons in which we may not have been able to detect such a relationship. Although no previous literature exists on T and surface area and estradiol and cortical change, the lack of current T and cortical thickness results may be limited by the current study’s statistical power. In fact, the effect sizes of testosterone on cortical thickness reported in a large sample (>200 participants) by Nguyen were relatively small (ρ = ~.2) [19]. Power analyses suggest that in order to detect similarly small effects with 80% power (α = .05), we would need a total sample size of 150 participants. Another possible reason we may have not been able to detect a significant effect of T is due to the distinct developmental trajectory of cortical thickness versus surface area [10]. That is, non-linear modeling in longitudinal samples with 3+ time points has shown steeper cortical thickness trajectories compared to surface area across development [10]. Thus, we were unable to capture a non-linear relationship between T and cortical thinning (as we had only 2 time points), but rather only linear changes in surface area in this sample. In fact, the longitudinal results previously reported by Nguyen and colleagues support the idea that T’s influence on cortical thickness may be non-linear, as relationships between T and thickness across the age range of 4 to 22 were driven by post-pubertal boys aged 14 and over, while no associations were observed in younger boys aged 4–13 [19].

In terms of E_2_, we found that individual differences in the *increases* in E_2_ predict greater thinning over time during adolescence. Notably, the current E_2_ findings were seen in the middle temporal gyrus, a region with distinct spatial-temporal patterns of E_2_ receptors across development and into adulthood (observed post-mortem)[39]. In cross-sectional studies, negative relationships were also found in portions of the temporal, frontal and parietal lobes [22] and cingulate and occipital gyri [21]. However, given our difficulty to precisely measure post-menarche girls in their follicular phase, these results should be interpreted with caution. Most of the girls in our sample began menstruation just prior to Time 2 data collection, and had irregular and highly variable cycles. This seems to be consistent with the literature, as menstrual diary studies show that variability in menstrual cycle length is greatest 1–2 years following menarche [32] and approximately 80% are often anovulatory in the first year after menarche [33,34]. The inability to standardize data collection at the same point in the menstrual cycle for each girl is likely to have led to the increase in variability in E_2_ values at Time 2, and also could have limited our power to detect certain effects. More research, with multiple estradiol measurements across irregular menstrual cycles in recent post-menarche girls, is needed to confirm the current findings as well as remove the possibility of this potential confound.

For the regions where individual differences in T and E_2_ contribute to the developmental trajectories of cortical thickness and surface area, the biological mechanism(s) may be both distinct and region-specific. Individual differences in T and E_2_ could contribute to either synaptic pruning or increases in myelination, or both, which could lead to changes in cortical thickness and surface area on T1-weighted MRI. T and E_2_ have been shown to be involved in neuronal differentiation, neuroprotection, neuronal survival and development [40,41]. However, animal studies have shown the impact of T and E_2_ on neural development to be tissue and dose specific[42] [43]. In terms of myelination, E_2_ and T have been found to differentially regulate oligo-progenitor cells, oligodendrocyte morphology, synthesis of basic myelin protein, and myelin formation [44,45]. Moving forward, joint MRI and histological research protocols using animal models may help us gain insight on the biological underpinnings of puberty-related cortical maturation as measured by MRI-based metrics.

Both sex-dependent and sex-independent effects were seen for physical markers of pubertal maturation. Both sexes showed that individuals with greater changes in physical maturation during the two-year follow-up had less superior frontal thinning, whereas less superior temporal thinning was only seen in girls. In addition to thickness, individual differences in physical changes predicted decreased surface area in the left precuneus in both sexes but also in the banks of the superior temporal sulcus in boys. Following the adolescent decline in thickness, adulthood is characterized by stable cortical dimensions [10,46,47]. Thus, larger changes in pubertal maturation (towards a sexually mature status) paralleled less cortical thickness change, suggesting pubertal development may contribute to achieving adult cortical phenotypes in distinct regions of the superior frontal and temporal lobes.

The striking differences in the results obtained using hormonal as compared to physical markers of puberty in the current study was not surprising to us. Tanner Stage does not necessarily show a 1-to-1 correspondence with E_2_ and T. Tanner staging is based on primary and secondary sex characteristics and is often used as a proxy of hormonal changes during puberty. Furthermore, physical changes during puberty may also, at the cellular level, reflect the sensitivity of an individual to hormone exposure as determined by genetic variations in hormone receptors [48]. Beyond T and E_2_, the additional sex steroid, progesterone, as well as pituitary protein hormones (luteinizing hormone (LH) and follicle-stimulating hormone (FSH)), adrenal androgens (such as DHEA), growth hormones and growth factors also show rapid increases to varying degrees as a function of pubertal maturation. Thus, some of the patterns found here between thickness and surface area and physical changes, as captured by Tanner Staging, may be driven by one or more of these hormonal processes during puberty. Although less research exists, a few cross-sectional studies have shown that LH levels relate to brain structure [21,49], including decreases in total gray matter volume in girls but not boys (although these effects did not remain significant after controlling for age) [21]. Furthermore, not only has DHEA been shown to relate to cortical thickness in portions of the frontal and parietal-temporal junction, but also there was an interactive effect for DHEA and T on thickness in regions of the cingulate and occipital pole [20]. Thus, more research is needed to determine which hormones, and possible interactions among them, may underlie individual differences in brain patterns of pubertal maturation as indexed by physical growth markers.

The current results should be considered in the larger context of the additional *within-subject* sex-hormone findings previously reported from the current longitudinal sample. That is, previous subcortical analyses performed on this longitudinal sample have shown significant associations between pubertal maturation (hormonal and Tanner Staging) and subcortical volumes [26], as well as between T and functional response to threat cues [24,25]. Large changes in subcortical volumes were seen during early puberty (as indexed by lower hormone levels), followed by smaller changes, or even a shift in growth, by late puberty [26]. Of particular interest was that increases in T predicted increases in amygdala volume in boys, but decreases in girls [26]. Increases in T over the two years also predicted increased amygdala activity [24], as well as decreased amygdala and orbitofrontal cortex coupling, to threatening stimuli [25]. In the current study, we expand on these findings to also show that individual differences in sex hormones also have associations with cortical thickness and surface area during these times. However, it is important to note, statistical analyses performed on this sample were different for subcortical volumes and cortical morphometrics (multi-level modeling vs. differences scores; the latter due to limitations of the Freesurfer’s Qdec software). Thus, while methodological differences limit our ability to directly compare the relationships seen between brain and hormones in this study, the data from this single longitudinal study as a whole, suggests that sex hormones influence both cortical and subcortical morphometric changes, as well as amygdala function during adolescence.

The following methodological limitations of the current study should also be noted. First, while this is one of the few studies to examine E_2_ and brain morphometry across adolescence, there are a number of challenges in accurately quantifying E_2_ given the circadian rhythms and cyclical changes that occur with the menstrual cycle. Moving forward, studies may consider menstrual diaries as well as measuring hormone levels at multiple time points within the month of imaging post-menarche girls to aid in reducing the possibility of this potential confound. Secondly, the current study may have been unable to detect more robust effects of hormone levels due to our small sample size. Thus, larger studies are needed to confirm the current findings in regards to the influence of sex hormones on brain development. Another statistical limitation to the current study is having only two time-points, which only allows for modeling a linear trajectory of development for each individual. Across adolescence, inverted U-shaped trajectories of age-related brain maturation are seen [50]. Unfortunately, three or more time points would be necessary to model non-linear trajectories of brain development that mirror a switch from growth to decline (e.g. peaking) in thickness and surface area. Thus, individuals who displayed rate of change values close to zero on cortical thickness and surface area metrics, may in fact have hit their “peak” between scans. Thus, future longitudinal research examining these factors will need to not only collect data across 3+ time points, but also should consider shorter scan-interval lengths, in order to completely rule out this potential problem.

In summary, the current study extends previous research by showing that physical and hormonal markers of puberty have small, region-specific associations with cortical maturation in girls and boys. Moreover, sex-specific *within-subject* pubertal changes predict frontal and temporal lobe thickness and surface area, which ultimately may contribute to sex differences in cognitive abilities, such as language and reasoning processing.

## Supporting Information

S1 Fig Within-subject changes in pubertal measurements. Raw data of within-subject changes in Tanner Stage, T, and E_2_ plotted by sex.
(TIF)
