# Supplementary figures and images for: A Longitudinal Study: Changes in Cortical Thickness and Surface Area during Pubertal Maturation
Source: PLoS One. 2015 Mar 20;10(3):e0119774. doi: 10.1371/journal.pone.0119774 (PMC4368209; doi:10.1371/journal.pone.0119774)

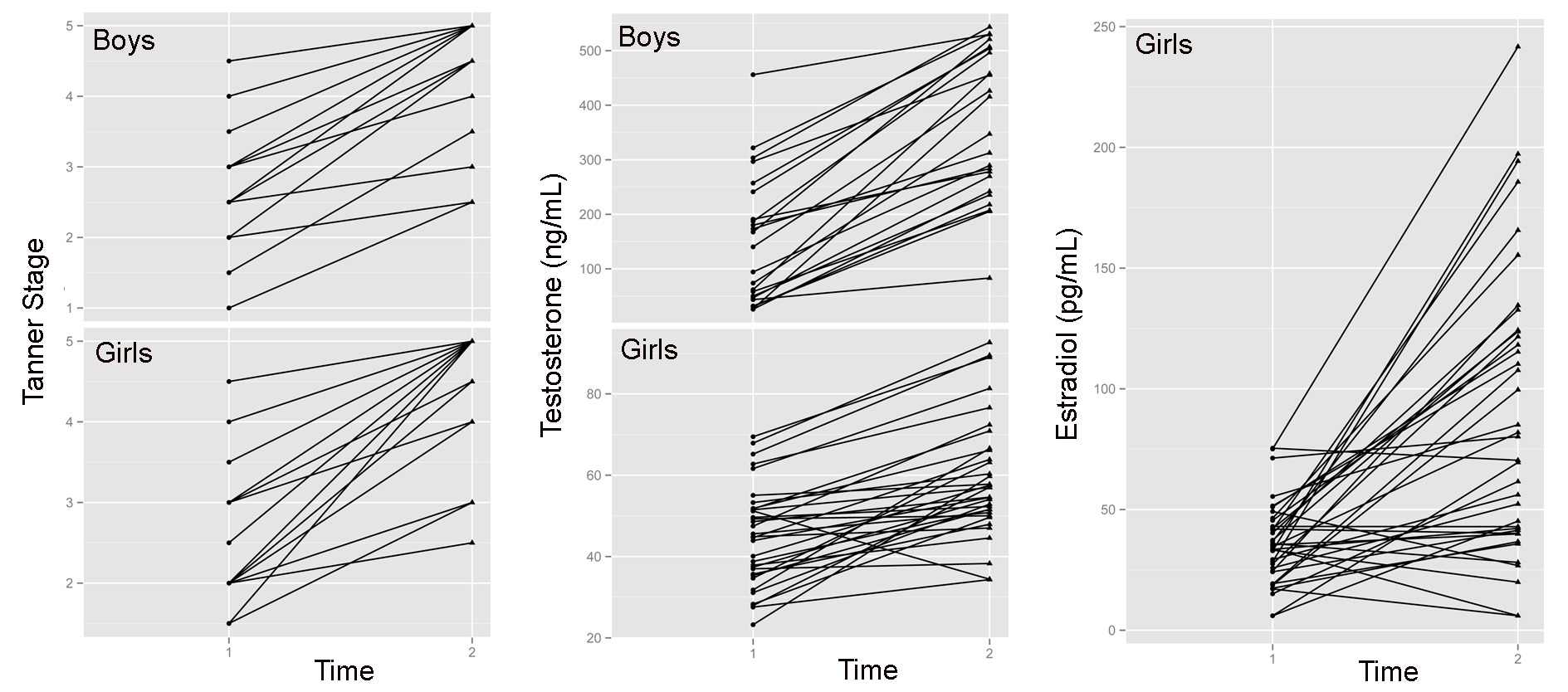

Supplement: S1 Fig — Raw data of within-subject changes in Tanner Stage, T, and E2 plotted by sex. (TIF) [file pone.0119774.s001.tif]
